# Current Status and Future Directions of Bacteria-Based Immunotherapy

**DOI:** 10.3389/fimmu.2022.911783

**Published:** 2022-06-10

**Authors:** Quan Tang, Xian Peng, Bo Xu, Xuedong Zhou, Jing Chen, Lei Cheng

**Affiliations:** ^1^ State Key Laboratory of Oral Diseases, National Clinical Research Center for Oral Diseases, West China Hospital of Stomatology, Sichuan University, Chengdu, China; ^2^ Department of Operative Dentistry and Endodontics, West China Hospital of Stomatology, Sichuan University, Chengdu, China; ^3^ Cancer Institute, Xuzhou Medical University, Xuzhou, China; ^4^ Center of Clinical Oncology, Affiliated Hospital of Xuzhou Medical University, Xuzhou, China

**Keywords:** immunotherapy, bacterial therapy, engineered bacteria, synthetic biology, microbiology

## Abstract

With the in-depth understanding of the anti-cancer immunity, immunotherapy has become a promising cancer treatment after surgery, radiotherapy, and chemotherapy. As natural immunogenicity substances, some bacteria can preferentially colonize and proliferate inside tumor tissues to interact with the host and exert anti-tumor effect. However, further research is hampered by the infection-associated toxicity and their unpredictable behaviors *in vivo*. Due to modern advances in genetic engineering, synthetic biology, and material science, modifying bacteria to minimize the toxicity and constructing a bacteria-based immunotherapy platform has become a hotspot in recent research. This review will cover the inherent advantages of unedited bacteria, highlight how bacteria can be engineered to provide greater tumor-targeting properties, enhanced immune-modulation effect, and improved safety. Successful applications of engineered bacteria in cancer immunotherapy or as part of the combination therapy are discussed as well as the bacteria based immunotherapy in different cancer types. In the end, we highlight the future directions and potential opportunities of this emerging field.

## 1 Introduction

In recent decades, the comprehensive cancer treatment including surgery, radiotherapy, and chemotherapy has improved the overall survival rate and quality of life for numerous cancer patients; however, intractable problems such as unforeseen side effects, inaccurate curative efficiency, and high recurrence tendency still exist, necessitating the development of better intervention strategies (1).

Immunotherapy which utilizes agents to reactivate or boost immune surveillance appeals to be a novel and promising strategy for cancer treatment in recent years (2). Some of the therapeutic drugs such as interferon-α (IFN-α) for hairy cell leukemia (3), interleukin-2 (IL-2) for metastatic renal cancer and metastatic melanoma (4) have been approved by the US Food and Drug Administration (FDA), and has achieved certain remission in some patients. However, the short therapeutic duration of IFN-α (5) and the high toxicity and relatively low response rate of IL-2 (6) were reported. In 2011, ipilimumab, a monoclonal antibody that bind to cytotoxic T lymphocyte antigen 4 (CTLA4), was approved for advanced melanoma (7), which introduced the significant immune checkpoint inhibitor and ushered in a new age of immunotherapy. A series of other checkpoints such as programmed cell death protein-1 (PD-1), programmed cell death-ligand 1 (PD-L1) and lymphocyte activation gene-3 (LAG-3) have also been identified to promote tumor immune escape and tumorigenesis. Therefore, inhibitors against these targets have been extensively developed and approved by the FDA for various cancer therapies, which significantly improved the survival rate of the advanced cancer patients in some clinical practices (8). However, the “cold” tumor microenvironment (TME) (9, 10) which is characterized by lacking of infiltrating immune cells or with exhausted immune cells compromises immune checkpoint blockade therapy and accounts for the non-responsiveness of some cancer patients, necessitating the development of improved immunotherapeutic strategies.

Tracing back to the origin of the modern immunotherapy, bacteria have been utilized as medication to treat incurable cancers. William Coley injected heat-inactivated *Streptococcus* and *Serratia marcescens* (known as Coley’s toxins) into malignant tissues and observed the ablation of sarcomas in the nineteenth century (11). Following further investigation, the researchers discovered that the toxins could trigger the activity of the immune system against tumors (12), thus William Coley was honored as the father of immunotherapy (13). Progressively, the interactions between bacteria and the immune system in the context of cancer has extensively developed the field of immunotherapy around the globe. A successful example, Bacillus Calmette–Guérin (BCG), which is a live attenuated strain of *Mycobacterium tuberculosis* variant *bovis* originally designed as a vaccine for tuberculosis (14), has been approved by the FDA for the treatment of bladder cancer (15). But further development of this biological therapy was stalled due to the infection-associated toxicity and the insufficient comprehension of tumor immunity at the time (16).

During the recent years, studies have also demonstrated the existence of intratumoral bacteria and the immune modulation roles of microbiota, indicating that the tumor tissue is a complex of bacteria interacting with tumor cells and the host (17). Bacteria involve into almost all biological aspects of cancer, though the effect is two-sided. Pathogens including *Helicobacter pylori, Fusobacterium nucleatum*, and *Staphylococcus aureus* can cause the chronic inflammation and contribute to the tumorigenesis (18–20). Probiotics and some certain species of bacteria can induce direct cell apoptosis which show promising characteristic to serve as anti-cancer preparations (21, 22). The recent study demonstrated the intracellular bacteria in breast cancer contributed the lung metastasis *via* the cytoskeleton remodeling which indicating that targeting the intracellular bacteria might be a therapeutic choice (23). Bacteria also involved into the anti-cancer drug metabolism, like chemotherapeutic drug gemcitabine was disintegrated by intratumoral bacteria in pancreatic ductal adenocarcinoma (24). Bacteria derived HLA-bound peptides showed immunogenic properties which could be further studied (25). These days, with the in-depth understanding of TME and the rapid advancement of microbiology, nanotechnology and recombinant DNA technology, reprograming bacteria and building genetic circuits that can control their behavior are now becoming conceivable, making bacterial therapy a new hotspot in current cancer research and treatment development (26–28). As the genome information of a large number of bacteria has been successfully deciphered, *Escherichia coli* and *Salmonella typhimurium (* 29), have evolved into highly editable engineered microorganisms that can be artificially endowed with diverse traits to facilitate them become sophisticated weapons against cancer.

This review will focus on the role of bacteria in anti-cancer immunity, as well as the present practice of employing bacteria as carriers or therapeutic agents in immunotherapy. The benefits of unmodified bacteria in immunotherapy will be discussed first, followed by engineered bacteria as enhanced treatment strategies. And the application of engineered bacteria in combined immunotherapy as well as the roles of bacteria-based immunotherapy in specific tumors are also discussed.

## 2 The Natural Advantages of Bacteria

Bacteria show tumor-targeting properties, and their surface structure or metabolites can also activate the immune system to exert anti-tumor effects. Therefore, bacteria are blessed with inherent advantages to function as therapeutic agent or carriers in tumor immunotherapy. This section will highlight the chemotaxis of bacteria to tumors and the immune activation effect.

### 2.1 Tumor-Targeting Properties of Bacteria

The vasculature in tumor tissue is generally chaotic and irregular, leading to insufficient diffusion of oxygen and nutrients (30). As a result, the central region of tumors is often presented as a hypoxic environment with necrotic tissues, where the oxygen pressure is as low as 7-28mmHg, while it is 40-60mmHg for normal tissues (31). Studies have found that this central area could provide a safe haven for some obligate and facultative anaerobes to colonize and proliferate after systemic administration (32). Zheng *et al.* reported that the number of *S. typhimurium* in the tumor site reached 1×10^10^CFU/g after intravenous administration for 3 days, and the ratio of tumor to normal organ bacteria exceeds 10000:1 (33). Shi *et al.* also found that *Bifidobacterium* could be detected inside the tumor sites one week after systemic administration, while remained undetectable in the lung (34). On the contrast, traditional chemotherapeutic drugs that solely rely on the passive distribution and limited permeability, are poorly accessible to these necrotic areas with sparse blood vessels through systemic administration, which leads to the relapse of tumors since the dormant but viable cancer cells still reside in the center zone (35). Therefore, bacteria are capable of colonizing the tumor core, the deepest and most difficult region to target for other types of agents.

The mechanism by which bacteria migrate to tumor sites remains to be fully elucidated. Some studies suggest that the disorganized vasculature in malignant tissues, preferential colonization and reproduction of bacteria in TME are the main factors endowing bacteria with tumor chemotaxis (36). When attenuated bacteria were injected intravenously, most of the bacteria were cleared by the oxygen-rich environment and immune cells in the physiological tissues, however, the motility of bacteria prompts them to cross the vascular system and disperse themselves to the hypoxic area in the center of the tumor, where the hypoxic environment and the nutrients released by the necrotic cancer cells promote the massive proliferation of the anaerobic bacteria. Meanwhile, the local immunosuppressive microenvironment also prevents them from being cleared in the early colonization stage (37), during which process, TNF-α and its induced hemorrhagic necrosis play an important role. Leschner *et al.* found that injection of *S. typhimurium* into tumor tissue increased TNF-α levels in circulatory system and induced increased local hemorrhage. As the bacteria flowed out of the blood vessels, they were trapped in the irregular vasculature, resulting in its colonization in tumors. When the researchers neutralized TNF-α in the blood, the blockage of blood flow and the reduction of bacterial colonization were observed (38), further verifying the role of TNF-α.

### 2.2 Immune Activation Properties of Bacteria

Hypoxia, as a hallmark for TME, also leads to the suppressive function of local immune cells (39, 40). With the tumor development, the uncontrolled proliferation of cancerous cells deprives the oxygen and nutrients from immune cells (41). The immune cells therefore tend to be exhausted, present a suppressive phenotype by secreting pro-cancer cytokines and chemokines and fail to respond the anti-cancer signals. However, bacteria derived molecules such as peptidoglycan, lipopolysaccharide (LPS), and lipoteichoic acid can provide strong immune stimuli signals. They mainly bind to pattern recognition receptors (PRRs) expressed by innate immune cells such as dendritic cells (DCs) and macrophages to induce significant migration of immune cells, stimulate the immune system to recognize and kill tumor cells (42). For instance, *Salmonella* LPS can increase the expression of IL-1β and exert the anti-tumor effect through the inflammasome and the Toll-like receptor 4 (TLR4)-mediated signaling pathway (43). As a structure of some Gram-negative bacteria, flagella can promote the expression of various pro-inflammatory cytokines, NO, H_2_O_2_, and chemokines by binding to Toll-like receptor 5 (TLR5) on dendritic cells (44), enhance the tumoricidal effect mediated by CD8^+^ T cells and down-regulate the suppressive function of Treg cells (45). Studies have shown that *Bifidobacterium* could stimulate stimulator of interferon genes (STING) and increases cross-priming of DCs (34). In addition to enhancing anti-tumor immunity by promoting the secretion of immune active factors, studies have shown that *Salmonella* can lead to up-regulation of connexin 43 (Cx43) expression in melanoma cells, mediating the formation of gap junctions between tumor cells and adjacent dendritic cells. Through this structure, tumor cells can present antigenic peptides to dendritic cells to activate the killing effect of cytotoxic T cells (46, 47). Si *et al.* also reported that oral administration of *Lactobacillus rhamnosus* GG increased tumor infiltrating DCs and promoted recruitment of CD8+ T cells through the type I IFN signaling.

Various cells such as macrophages and myeloid-derived suppressor cells (MDSCs) play important roles in the formation of the immunosuppressive microenvironment, which represents a therapeutic regimen for manipulating these cells to reverse the suppressive TME (48, 49). Certain components of bacteria can mediate the phenotypic transformation of immune cells. For example, macrophages make up a considerable percentage of immune cells and play an important role in immune regulation. According to their surface chemicals and functionalities, they are split into two subtypes. Anti-tumor macrophages mediate phagocytosis, release pro-inflammatory cytokines, whereas pro-tumor macrophages secrete anti-inflammatory cytokines, mediate tumor angiogenesis (50). Studies have found that flagellin can mediate the transformation of pro-tumor macrophages to anti-tumor macrophages, transforming the immunosuppressive microenvironment into an immunocompetent environment (33). Researchers has also reported that a variety of *Lactobacillusi* species promoted anti-tumor M1-like polarization through the TLR2 signaling pathway (51, 52). In addition, MDSCs exist in the blood of cancer patients and have a strong inhibitory effect on T cells and NK cells. Studies have found that *Listeria* can infect MDSCs, reduce the content of MDSCs in the blood, and promote the remaining MDSCs to secrete IL-12, switching to an immunocompetent phenotype (53). In addition, a reduction in tumor growth was observed in animal models treated with *Listeria*, suggesting that *Listeria* can inhibit tumors by acting on MDSCs.

## 3 Engineering Bacteria for Therapeutic Improvement

**Figure 1:**
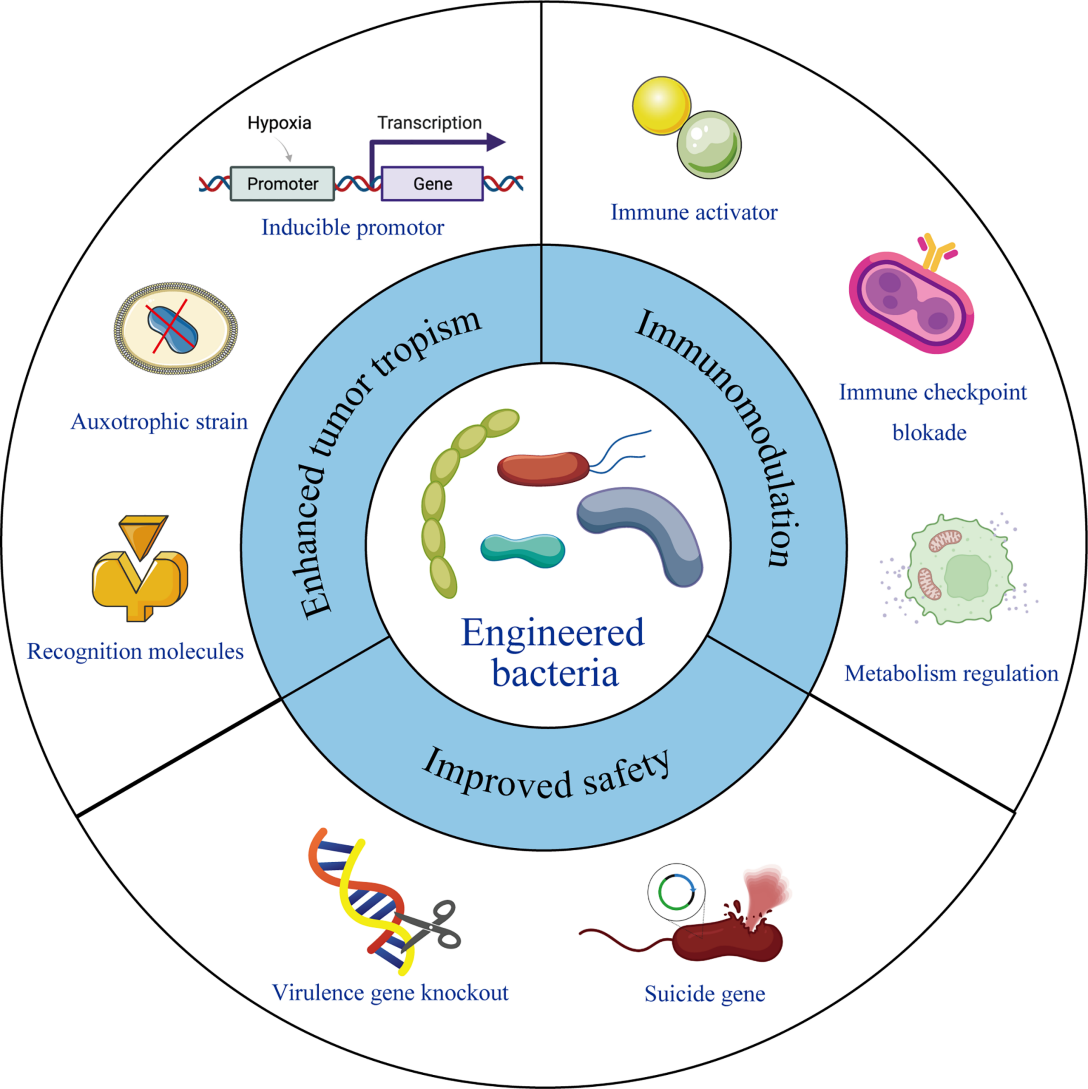
Engineering bacteria for therapeutic improvement. Under modern microbiology, nanotechnology and recombinant DNA technology, bacteria can be engineered with enhanced tumor tropism, significant immunomodulation and improved safety profile, leading to reformed therapeutic outcome.

The chemotactic colonization of bacteria at tumor sites, as well as their immunogenicity, makes them ideal candidates for immunotherapy. It has been reported that several bacteria were detected inside the tumor tissues and intratumoral delivery of probiotics can promote the anti-tumor immunity (34, 54), providing a theoretical foundation for the use of microbes in tumor treatment. In recent years, with the development of synthetic biology, material science and gene editing tools, bacteria engineering has become possible. The tumor targeting properties, therapeutic effects, and safety performance can be further improved by different ways of modifying and transforming. Following studies listed in **Table 1** and also shown in **Figure 1**, summarizes excellent prospects of engineered bacteria with 3 aspects of improved properties.

**Table 1:** Engineered bacteria for the enhanced therapeutic outcome.

Improvement	Strain	Mechanism	Cancer model	Ref
Enhanced tumor tropism	*S. typhimurium* A1	Leu/Arg-dependent auxotrophy	PC-3 human prostate cancer	(55)
	*S. typhimurium* SF104	Mutant of *aroA* gene	CT26 mouse colon cancer RenCa mouse renal cancer	(56)
	*E. coli* Nissle 1917	Thymidine and diaminopimelic acid auxotrophy	B16-F10 mouse melanoma EL4 mouse T-cell lymphoma A20 mouse B-cell lymphoma 4T1 mouse breast cancer CT26 mouse colon cancer	(57)
	*S. typhimurium* YB1	Place *asd* gene under a hypoxia conditioned promoter	MDA-MB-231 human breast cancer	(58)
	*S. typhimurium* VNP20009	Express CEA-specific antibody	MC38 mouse colon cancer	(59)
	*S. typhimurium* SL3261	Express CD20-targeting antibody	B16-F10 mouse melanoma MCA203 mouse fibrosarcoma CT26 mouse colon cancer Namalwa or Karpas299 human lymphoma	(60)
	*S. typhimurium* ΔppGpp	Display peptides that bind to αvβ3 integrin	MCF7, MDA-MB-231 human breast cancer MDA-MB-435, M21, M21L human melanoma U87MG human glioblastoma ASPC-1 human pancreatic cancer CT26 mouse colon cancer 4T1 mouse breast cancer MC38 mouse colon cancer	(61)
	*L. lactis* NZ9000	Display the binding protain of EpCAM and HER2	/	(62)
	*S. typhimurium* VNP20009	Bind aptamers to the bacterial surface	4T1 mouse breast cancer H22 mouse hepatocellular carcinoma	(63)
Immune modulation	*S. typhimurium* VNP20009	Production of IL-18	CT26 mouse colon cancer D2F2 mouse breast cancer	(64)
	*S. typhimurium* BRD509	Production of IFN-γ	B16-F10 mouse melanoma	(65)
	*L. lactis* NZ9000	Production of anti-CTLA-4 single chain fragment variable	/	(66)
	*E. coli* Nissle 1917	Production of STING-agonist cyclic di-AMP	B16-F10 mouse melanoma EL4 mouse T-cell lymphoma A20 mouse B-cell lymphoma 4T1 mouse breast cancer CT26 mouse colon cancer	(57)
	*E. coli* Nissle 1917	Production of PD-L1 and CTLA-4 nanobodies	CT26 mouse colon cancer A20 mouse B-cell lymphoma	(67)
	*E. coli* Nissle 1917	Production of PD-L1 and CTLA-4 nanobodies in a thermal sensitive manner	A20 mouse B-cell lymphoma	(68)
	*E. coli*	Production of nanobody antagonist of CD47	A20 mouse B-cell lymphoma 4T1 mouse breast cancer B16-F10 mouse melanoma	(69)
	*E. coli* Nissle 1917	Increase intratumoural concentrations of L-arginine	MC38 mouse colon cancer	(70)
	*S. oneidensis* MR-1	Reduce intratumoural concentrations of lactate	CT26 mouse colon cancer	(71)
Improved safety	*S. typhimurium* VNP20009	Deletion in the *msbB* and *purI* gene	B16-F10 mouse melanoma LOX human melanoma DLD-1 human colon cancer	(72)
	*S. typhimurium* ΔppGpp	Deletion in the *relA* and *spoT* gene	/	(73)
	*L. monocytogenes* 10403S	Mutant of *PrfA* gene	/	(74)
	*L. monocytogenes* ΔactA/ΔinlB	Deletion in the act*A* and *inlB* gene	CT26 mouse colon cancer	(75)
	*E. coli* Nissle 1917	Dynamic and tunable regulation of the bacterial surface	CT26 mouse colon cancer 4T1 mouse breast cancer	(76)
	*S. typhimurium* SL1344	Incorporation of synchronized lysis circuit	MC26 mouse colon cancer	(77)
	*E. coli* Nissle 1917	Integrate synchronized lysis circuit into genome	CT26 mouse colon cancer A20 mouse B-cell lymphoma	(67)

### 3.1 Engineered Bacteria Improve Tumor Tropism

It is critical to take the tumor-targeting abilities into consideration when designing therapeutic agents, which accounts for not only the healing effect but also the elimination of off-target damage. When it comes to the improvement of tumor tropism of engineered bacteria, it may be just as crucial to hinder their survival in normal tissues as it is to boost their accumulation in tumor sites.

#### 3.1.1 Construction of Auxotrophic Strain and Inducible Promoter

The construction of auxotrophic mutants is a strategy to improve bacteria targeting property. Based on the difference of nutrients contained in normal tissues and tumor sites, mutants can be designed to be only able to colonize and survive in tumor tissues. Among them, Salmonella A1 and SF104 are examples of successful application. Salmonella A1 is an auxotrophic strain of leucine and arginine (55), while Salmonella SF104 shows the need for aromatic amino acids with the mutation of the gene *aroA (* 56), both of which can make the bacteria unable to enrich in normal tissues, but can specifically accumulate in tumor sites. *E. coli* Nissle was also designed by Leventhal *et al.* to include two auxotrophies (*thy*A and *dap*A) which result in its inability to survive outside the TME and in its inability to reproduce within the TME, respectively (57).

The essential gene *asd* of Salmonella, which mediates the synthesis of diaminoacrylic acid (DAP), an important component of the cell wall of Gram-negative bacteria, is placed under a hypoxia-inducible promoter by Yu *et al.* In normal tissues, the synthesis of DAP is blocked, without the supply of exogenous DAP, the bacteria will be lysed. However, the gene *asd* can be expressed in tumor sites with hypoxic environment, which enables Salmonella to colonize and survive in tumors. To further reduce off-target effects, they also placed the expression of inhibitory antisense RNA against *asd* under an aerobic-inducible promoter, and finally the strain showed 1000-fold enrichment in tumor sites compared to other organs (58). In addition, exogenous substances or stimuli, such as L-arabinose (78), acetylsalicylic acid (79), radiation stimulation (80), etc., can also regulate the expression of essential bacterial genes under the corresponding inducible promoters, which is beneficial to ensure specific proliferation at the tumor sites.

#### 3.1.2 Modification Tumor-Related Recognition Molecules

Engineering synthetic adhesins tailored to bind specified cancer-expressed molecules such as neoantigens or other molecules abundant in cancer cells can improve some bacteria’s natural affinity for tumors. Bereta *et al.* observed increased bacterial aggregation at tumor sites by expressing a specific single-chain antibody fragment for carcinoembryonic antigen (CEA) on *S. typhimurium* VNP20009 (59); Massa *et al.* increased bacteria’s invasiveness against CD20^+^ lymphoma, while reducing non-specific aggregation by binding anti-CD20 antibody to the surface of Salmonella (60). αvβ3 integrin is overexpressed in a variety of malignant tumors. By fusing arginine-glycine-aspartate peptides to bacterial outer membrane protein A, Park *et al.* enabled the bacteria to specifically bind to αvβ3 integrins and observed significant antitumor effects in xenogeneic melanoma and breast cancer transplant models (61). Epithelial cell adhesion molecule (EpCAM) and human epidermal growth factor receptor 2 (HER2) are transmembrane glycoprotein receptors associated with colorectal cancer. Plavec *et al.* successfully observed the co-localization of bacteria and tumor cells by displaying the binding protein of EpCAM and HER2 on the surface of *Lactococcus lactis* and making the bacteria express the infrared fluorescent protein for imaging, while on cells that did not express the corresponding molecule, no bacterial binding was observed (62). In addition, an aptamer is an oligomeric nucleic acid that can specifically bind to a certain molecule and has similar ligand-receptor binding characteristics with the target molecule. By binding the aptamer AS1411 to the surface of *S. typhimurium* VNP20009, Geng *et al.* observed nearly 2-fold and 4-fold enrichment after 12 and 60 hours in 4T1 and H22 tumor-bearing mouse models compared to unmodified bacteria, showing the enhanced targeting performance of this bacterium (63).

### 3.2 Engineered Bacteria Regulate the Immune Microenvironment

It has been stated that several fundamental components of bacteria are able to alter the immune system of the human body. However, to obtain greater immune regulatory effects, the engineered bacteria can be designed to load or express exogenous immunotherapeutic medications for enhanced anti-tumor efficacy.

#### 3.2.1 Delivery of Immune-Activating Agents

Given that bacteria preferentially colonize malignant regions and naturally stimulate innate immune cells, bacteria-based therapy can provide a baseline level of immune activation in tumor tissues. Immune activators can effectively reform the immunosuppressive microenvironment of tumors and are one of the commonly used therapeutic agents for immunotherapy, which mainly include cytokines, tumor antigens and other substances. Cytokines own the ability to promote the activation and proliferation of immune cells, and the delivery of cytokines through engineered bacteria are blessed with the characteristics of high specificity and low side effects. Loeffler *et al.* used attenuated *S. typhimurium* to synthesize IL-18. By increasing the infiltration of CD3^+^/CD4^+^ T cells and DX5^+^ NK cells in the tumor area, the expression of cytokines such as IL-1β, TNF-α, IFN-γ, GM-CSF were increased, and the anti-tumor effect was also observed (64). Yoon *et al.* also genetically modified Salmonella to express and secrete IFN-γ, thereby activating NK cells and mediating direct killing of cancer cells (65). Stimulator of interferon genes (STING) is another immune activating agent that can initiate tumor-specific T cell responses by activating antigen-presenting cells, producing type I interferons, and mediating antigen cross-presentation to cytotoxic T cells (81). Leventhal *et al.* expressed the STING agonist cyclic adenosine diphosphate through non-pathogenic *E. coli* Nissle, and observed the expression of type I interferon and various proinflammatory cytokines such as TNF-αIL-6IL-1βGM-CSF were up-regulated after intratumorally injection (57). The strain caused robust tumor eradication and long-term immunological memory in mice with tumors that were sparsely infiltrated by T cells, making treated mice resistant to tumor relapses. Tumor antigens are often used to make tumor vaccines to enhance immunity and activate immune cells to kill cancer cells. Tumor vaccines using bacteria as carriers have also been vigorously developed, exhibiting promising application prospects (82, 83). The human papillomavirus type 16 oncoprotein E7 (HPV-16 E7) plays a key role in the pathogenesis of cervical cancer and is required for host cell immunization. It is reported that oral administration of *L. lactis* expressing HPV-16 E7 protein could lead to significant delay of E7-expressing tumor growth, with significant increase in the numbers of E7-specific CD4^+^T helper and CD8^+^T cell, indicating that this bacteria-based vaccine provided profound protective effects against tumor cell challenge (84). A phase I clinical trial of this oral vaccine is also underwent to verify its safety and immunogenicity (85).

#### 3.2.2 Delivery of Immune Checkpoint Inhibitors

Immune checkpoint therapy has been approved by FDA for the treatment of clinical cancer patients, and has achieved certain clinical results in the treatment of melanoma (7), non-small cell lung cancer (86), etc. The main mechanism of these drugs is to block the immunosuppressive state mediated by cancer cells and relieve the immune tolerance state (87). Monoclonal antibodies against PD-1, PD-L1 and CTLA4 have been widely used. Namai *et al.* successfully expressed human anti-CTLA4 antibody in *L. lactis* by genetic modification, and confirmed its recognition and binding to human CTLA4 by ELISA (66). Gurbatri *et al.* also used a combination of anti-PD-L1 and anti-CTLA4 therapy. The team transformed high-copy plasmids carrying anti-PD-L1 antibodies and anti-CTLA4 antibodies into engineered *E. coli* to achieve controllable expression of PD-L1 and CTLA4 antagonists in tumor sites. And the decrease in the number of Treg cells and the increase in the number of CD4^+^ and CD8^+^ T cells have been observed through immunophenotyping studies, indicating that the immunosuppressive microenvironment at this site has been reversed (67). To achieve the selective release of therapeutic agents at the tumor regions, Shapiro *et al.* further manufactured strains that produced tumor-suppressing anti-PD-L1 and anti-CTLA4 antibodies only when heated to a trigger temperature of 42-43°C by introducing a temperature-actuated genetic state switch. Since the normal human body temperature is 37°C, these strains do not express anti-tumor nanobodies after systemic administration. Instead, they grow inside tumors until a triggering temperature is reached by the utilization of focused ultrasound (68).

CD47 is an anti-phagocytic receptor that overexpressed in multiple cancer types. Chowdhury *et al.* delivered anti-CD47 antibodies by engineering *E. coli* to activate dendritic cells in the TME and increase the phagocytosis of cancer cells, which also promoted the cross-presentation of tumor antigens, activated infiltrating T cells, and achieved rapid tumor regression (69).

#### 3.2.3 Regulation of Metabolic Pathways of Tumor Immune Cells

L-arginine is critical for anti-tumor T cell responses (88), yet low availability of L-arginine in malignant tissues contributes to low T cell responses and the poor efficacy of immune checkpoint inhibition therapy. The Canale team found that the local concentration of L-arginine could not be maintained by injecting a saturated solution of L-arginine into the tumor, so the team leveraged engineered *E. coli* Nissle 1917 to continuously convert the metabolic waste ammonia into L-arginine in the tumor sites, which effectively increased the intratumoral L-arginine concentration and enhanced the T cell response. And a synergistic effect with anti-PD-L1 therapy was also observed, exerting a stronger antitumor effect (70). As studies has revealed that lactate could be responsible for tumor invasion (89), targeting lactate metabolism is a feasible therapeutic strategy. Chen *et al.* fabricated a biohybrid material with significant lactate exhaustion property, in which manganese dioxide nanoflowers as electron receptor was modified onto the surface of *Shewanella oneidensis*. Therefore, the extracellular lactate serves as electron donor to ensure a sustained effect of downregulating the lactate level by the coupling of bacterial respiration with tumor metabolism, which result in inhibited tumor progression (71).

### 3.3 Engineered Bacteria Improve Safety

Although bacteria exhibit excellent anti-tumor characteristics, their potential toxicity is a major stumbling block to their application. The safety profile of living bacteria preparations, on the other hand, represents a crucial need for their clinical translation. To make full use of bacteria to fight against cancer, researchers have made tremendous efforts to construct a large number of attenuated engineered strains to improve their safety performance.

#### 3.3.1 Virulence-Related Gene Knockout

The immunogenic bacterial surface molecule contribute as main virulence of bacteria, which indicates that modification (such as genetic knockout) of these surface antigens represent a major approach to circumvent toxicity of living pathogen. For instance, ppGpp (guanosine 5’-diphosphate-3’-diphosphate) is a signaling molecule involved in the expression of virulence genes. By knocking out the *relA* and *spoT* genes, the synthesis of ppGpp was blocked, resulting in a 10^5^-10^6^-fold increase in its LD50 compared to wild strains (73). The bacteria with disordered ppGpp synthesis also showed good antitumor activity due to its ability to induce the secretion of pro-inflammatory factors IL-1β, IL-18, and TNF-α (37). In addition, LPS of Gram-negative bacteria is a potent stimulator for inducing TNF expression and is one of the main causes of sepsis. On the other hand, as the outermost structure of the cell envelop, LPS is also an important barrier and defense agent and is essential for their survival and efficient tumor colonization. VNP20009 is a safe strain of *Salmonella* with deletion of *msbB* and *purI* genes (72), in which *msbB* knockout leads to myristoylation of lipid A in LPS, reducing the ability to induce TNF secretion and greatly reducing its virulence (90). However, the structural changes of lipid A also reduced its therapeutic effect. In clinical trials, the tumor colonization and antitumor activity in human patients were not effectively exhibited (91), suggesting the apparent trade-off between bacterial virulence and antitumor activity. To maintain the balance between safety and anti-tumor efficacy, Frahm *et al.* observed that the attenuated bacteria exerted good therapeutic effects by integrating the LPS biosynthesis gene into the araBAD locus of the bacterial chromosome with the regulation of arabinose-inducible promoter (92). To step further, Harimoto *et al.* realized a dynamic and tunable regulation of the bacterial surface by constructing an inducible synthetic gene circuit that modulates the programmed expression of bacterial surface capsular polysaccharide. In this way, the bacterial surface virulence molecular is hided and shielded from the immune system, which turn out to show enhanced bacterial survival and colonization and a ten-fold increase in systemically injectable tolerated dose *in vivo*, showing an improved safety profile (76).

In addition to modifying virulence molecules, aiming at the escape ability and invasiveness of bacteria is also a major measure for attenuation. *Listeria* is a vaccine strain mainly used to express tumor antigens, whose virulence factor can be deleted by knocking out the *prfA* gene (74). Unfortunately, in this way, *Listeria* cannot escape from the phagosome, which prevents the carried tumor antigens from entering the cytoplasm for processing. To address this issue, the strain was designed to express low levels of PrfA and *Listeria* hemolysin O to improve its immunogenicity, which showed that the reformed strain is endowed with great potential in expressing tumor antigens as well as delivering other therapeutic drugs (93). CRS-207, a *Listeria* strain with two virulence genes *actA* and internalin B knocked out, exhibited reduced spreading and invasive abilities. And its colonization level decreased by 1000 times compared to common strains (75), thus the application security is guaranteed.

#### 3.3.2 Suicide Gene

To avoid the infinite proliferation of bacteria in the body, strategies must be adopted to programmatically limit the proliferation level to maintain the stability of the microecology. Din *et al.* designed a synchronized lysis circuit (SLC) into which a phage *φ*X174 cleavage gene E was integrated. This method takes advantage of the colony effect of natural bacteria. When the bacterial proliferation reaches a threshold density, the bacteriophage-derived lysis factor is produced, diffuses to neighboring cells and triggers lysis. This releases the intracellular therapeutic drugs, while a small number of surviving bacteria continue to reproduce to maintain the dynamic balance of local bacterial populations (77). Still, a major disadvantage of this approach is its dependence on plasmids, which may lead to recombination, mutation and loss during the growth cycle. As to make the circuit more stable, Gurbatri *et al.* integrated the gene circuit into the genome of *E. coli*. Although a certain number of copies of quorum sensing genes was lost, the results showed that this method has better effect than the original system (67).

## 4 Application of Engineered Bacteria in Combined Immunotherapy

It has been shown that bacteria can function as immunotherapeutic agents to enhance the anti-tumor immunity. As combination therapy is a widely used strategy to improve the overall effect, bacteria-based immunotherapy has also been served as a part of combination with chemotherapy, radiotherapy, photodynamic therapy and photothermal therapy. In this section, we summarize the latest practices of bacteria being recruited as part of the combined therapy, where bacteria exhibited synergistic effect of activating the immune system, synthesizing or protecting the anti-cancer drugs to enhance anti-tumor effect.

### 4.1 Combined With Chemotherapy

Traditional chemotherapy suffers from a lack of specific delivery to malignant tissue and significant drug systemic exposure, which commonly results in dose-limiting toxicity. Applying engineered bacteria to act as drug delivery system for controlled drug release, as well as utilize their immunogenicity for immune modulation has gained much research attention. Ektate *et al.* attached low-temperature sensitive liposomes onto the membrane of *Salmonella*, which mediated the triggered release of doxorubicin inside colon cancer cells with the help of high intensity focused ultrasound (HIFU) heating, resulting in efficient drug delivery in both the cytoplasm and the nucleus of cancer cells. Moreover, the strain also polarized macrophages to anti-tumor M1 phenotype, enriched Th1 cells population with high production of TNF-α, and decreased expression of IL-10, thus exhibiting enhanced therapeutic effects in a combined chemo-immunotherapy manner (94). For some highly malignant tumor types, chemotherapeutic drugs alone show limited enhanced survival benefits, such as gemcitabine for the treatment of pancreatic ductal adenocarcinoma, thus calling for additional approaches. To reform the poorly immunogenic TME of pancreatic ductal adenocarcinoma, Gravekamp *et al.* delivered tetanus toxoid protein, which act as a neoantigen reactivating preexisting memory T cells that were generated during childhood vaccinations, into tumor cells by attenuated *Listeria*, which could selectively delivered to tumor regions with the help of MDSCs (53). The tetanus toxoid induced attraction of CD4 T cells, with increased production of IFNγ, perforin, and granzyme B in the TME, while gemcitabine was used to reduce immune suppression in the TME, which resulted in reduced tumor burden by 80% compared to untreated mice (95).

Besides leveraging living bacteria, bacterial outer membrane vesicles (OMVs), which are naturally produced from Gram-negative bacterial membranes during growth process, have recently emerged as immunotherapeutic agents for a variety of biomedical applications. Chen *et al.* encapsulated drug-loaded polymeric micelles into bacterial outer membrane vesicles, where the bacterial component could activate the immune response while the loaded tegafur exert both chemotherapeutic and immunomodulatory effect to ablate cancer cells. As a result, this strategy showed substantial improvement in tumor regression, survival extension and remarkable inhibition of pulmonary metastasis (96).

### 4.2 Combined With Radiotherapy

Bacterial-assisted radiotherapy represent as a new approach for tumor treatment. Although few studies has applied bacteria to improve radiotherapy, this field might be developed as a new viable method in clinical radiation oncology. In a study by Jiang *et al.*, the therapeutic effect of combining *E. coli* with radiotherapy was investigated, which revealed significant tumor shrinkage in a colon tumor model under 21 Gy of radiation and *E. coli* with the production of cytolysin A (97). Similarly, engineered *S. typhimurium* carrying imaging probes and therapeutic agents for tumor imaging and treatment in a combination of radiotherapy demonstrated greater remission. As a result, the bacteria carrying cytolysin A combined with radiotherapy cause more tumor remission as compared to bacterial therapy alone (98). In a recent study, an integrated nanosystem for sensitizing radiation was established using modified *E. coli* and Bi_2_S_3_ nanoparticles. The bacteria might invade tumor locations and overexpress the cytolysin A protein to switch the cell cycle from a radioresistant to a radiosensitive state. At the same time, Bi_2_S_3_ nanoparticles may improve radiation sensitivity by causing intracellular production of reactive oxygen species (ROS) and DNA damage (99).

After radiotherapy, tumors release a considerable number of tumor antigens, which can be taken up and presented by DCs, leading to specific adaptive immune responses. However, in the immunosuppressive TME, the number of DCs is typically low and they are usually remaining in a state of dysfunction, which indicate that intratumoral antigens are often poorly recognized and presented. As a result, increasing the number of DCs and boosting their function in tumors are major study topics. Wang *et al.* injected *Salmonella* coated with antigen-adsorbing cationic polymer nanoparticles into tumor tissues, which can capture the antigen released after radiotherapy and transport them out of the tumor core to activate the surrounding DCs in tumor marginal tissues owing to the bacteria’s mobility. As a result, large increases in activated DCs *in vitro* and extended survival in multiple tumor mice models *in vivo* were observed, showing the enhanced systemic antitumor effects (100).

### 4.3 Combined With Photodynamic Therapy and Photothermal Therapy

As standard tumor therapies suffer from unspecific killing effect and complicated surgery, photodynamic therapy and photothermal therapy have emerged as new therapeutic options due to their non-invasiveness, high specifity and excellent spatial and temporal control. Recently, numerous studies have attempted to employ bacteria as carrier to load the therapeutic agents of PDT and PTT, in order to leverage the tumor-targeting and immunoactivating properties of bacteria.

PDT relies on the conversion of local oxygen molecules into ROS to mediate the killing effect on cancer cells. But the local hypoxic environment of the tumor causes insufficient production of ROS, thus compromising the therapeutic effect of PDT. Liu *et al.* integrated photosensitizer-coated nanoparticles onto the surface of photosynthetic bacteria *Synechococcus*. Under 660nm laser irradiation, photosynthetic bacteria continued to produce oxygen, which ensured the production of ROS and enhanced the effect of photodynamic therapy. *Synechococcus*, as immunogenic bacteria, also activate local immunity by upregulating the expression of MHC class II molecules and IL-12. At the same time, this treatment method induces immunogenic apoptosis by up-regulating calreticulin on the cell surface, and has shown a good therapeutic effect in triple-negative breast cancer model (101).

Other researchers have also tried to combine bacteria with PTT. Indocyanine green (ICG) was bound to the surface of *S. typhimurium* strain YB1 by Liu *et al.* This stratery resulted in a 14-fold increase of the enrichment of the modified strain within tumors, as well as perfect photothermal conversion. In addition to significantly killing the tumor in the central hypoxic area, this method also effectively kills tumor cells in the peripheral area with normal oxygen perfusion, showing better anti-tumor efficacy (102). Chen *et al.* integrated the photothermal agent polydopamine on the surface of *Salmonella* and observed that the engineered bacteria exhibited unaffected tumor-targeting ability and activated local immunity by promoting the production of TNF-α and IL-4 (103). The research team further improved the strategy and realized an innovative triple therapy by combining the immune checkpoint inhibitor AUNP-12 (an anti-PD-1 peptide). Through the application of phospholipid phase separation gel, the team improved the short retention time of the peptide antagonist AUNP-12, and achieved a sustained release effect of the therapeutic drug at the tumor site for up to 42 days. This triple therapy showed a more pronounced antitumor effect than bacterial therapy alone and showed potent inhibition of advanced melanoma (104). However, these studies require the multistep synthesis of nanoparticles and complicated genetic manipulations of bacteria. Reghu *et al.* established a simple modification method by designing nanoparticle-functionalized nonpathogenic natural bacteria. To be more specific, they engineered *Bifidobacterium bifidum* with ICG-encapsulating Cremophor EL nanoparticles by simple incubation and washing processes while maintaining the bacterial natural properties. Under near infrared light induction, the functionalized bacteria showed superior antitumor effect by laser-driven photothermal conversion and the excess TNF-α expression with the assistance of macrophages (105). Similarly, Yang *et al.* apply non-pathogenic natural purplep synthetic bacteria *Rhodopseudomonas palustris* in cancer theranostics without complicated chemical functionalization and genetic manipulation, which are blessed with tumor-targeting abilities, excellent heat and ROS production, resulting in drastic tumor elimination (106).

## 5 The Role of Bacteria-Based Immunotherapy in Different Cancer Types

Different types of cancer have their unique biological behaviors, their response to the immune modulation also varies. Here, we summarized the immune related bacteria therapies according to their application in different cancer types. The therapeutic agents and the effect on tumor and the microenvironment were discussed in details, which were also summarized in **Table 2**.

**Table 2:** The role of bacteria-based immunotherapy in different cancer types.

Cancer type	Bacterium	Immune modulation effects	Ref
Colon cancer	*L. rhamnosus*	Restore the antibiotic-disrupted gut microbiota and synergize with anti-PD-1 therapy	(107)
	*Bifidobacterium*	Facilitate anti-CD47 therapy *via* STING signaling	(34)
	*L. acidophilus*	Improved serum levels of IFN-γ, IL-10, CD4^+^ and CD8^+^ cells	(108)
	*S. typhimurium*	Reduce intratumoral levels of IDO, increase tumor infiltration of neutrophils	(109)
	*S. typhimuriumS*	Inhibition of Stat3 combined with siRNA against PD-1	(110)
Lung cancer	*L. monocytogenes*	Enhanced function of CD8+ T cell and regulation effcets on Treg cells and MDSCs	(111)
	*B. bifidum*	Increased secretion of IFN-γ and IL-12, enhanced lymphocyte proliferation and CD8^+^ T cell responses	(112)
	*L. casei*	Increased production of IL-2	(113)
	*L. lactis*	Recombinant strain with IL-17A cytokine secretion	(114)
Melanoma	*L. monocytogenes*	Increased infiltration of CD4^+^ and CD8^+^ T cells	(115)
	*L. monocytogenes*	Elicit profound CD8^+^ T cells responses and synergize with immune checkpoint blockade	(116, 117)
Breast cancer	*S. typhimurium*	Elevated percentage of CD3^+^CD4^+^ T cells and increased production of IFN- γ and TNF-α	(63)
	*E. coli*	Local delivery of CD47 antagonist and activation of tumor-infiltrating T cells	(69)
Lymphoma	*E. coli*	Local delivery of PD-L1 and CTLA-4 nanobodies	(67)
	*E. coli*	Local delivery of CD47 antagonist and activation of tumor-infiltrating T cells	(69)
Prostate cancer	*S. typhimurium*	Induce Th1 immune responses and tumor protective immunity	(118)
Cervical cancer	*L. monocytogenes*	Induction of Th1 immunity, enhanced lymphocyte proliferation and specific CTL activity	(119)
Pancreatic cancer	*L. monocytogenes*	Reactivate the preexisting memory T cells by delivery of tetanus toxoid	(95)

### 5.1 Colon Cancer

The colon cancer is considered to be highly associated with the gut microbiota (120). Nowadays, emerging studies have demonstrated that the dysbiosis of gut microbiome poses adverse effects on the epithelial cells and eventually lead to the induction of colon cancer. Therefore, probiotics, which specifically suppress the colonization of certain pathogenic bacteria and reverse the dysbiosis of gut microbiome caused by antibiotic usage, have been reported to maintain the balance of intestinal microbiota and exert preventive effects against colon cancers (121). Sun *et al.* reported that oral administration of *L. rhamnosus* Probio-M9 could modulate the gut microbiota in which the relative abundance of beneficial bacteria was increased, and contributed to the recovery of antibiotic-disrupted gut microbiota. Moreover, synergistic effect of this probiotic therapy was discovered when coupled with the anti-PD-1 treatment, in which significant tumor inhibition was observed as compared to the anti-PD-1 treatment alone (107). Similarly, Fu *et al.* also found that intratumoral accumulation of *Bifidobacterium* facilitated anti-CD47 therapy *via* STING signaling (34). These studies pose valid evidence to support that the outcome of immune checkpoint blockade therapy relies on the host’s gut microbiota (122). Probiotics are also blessed with protective effects against the tumorigenesis. In an orthotopic colon cancer model induced by azoxymethane, oral intake of *L. acidophilus*, and *B. bifidum* probiotics were reported to inhibit the colon lesions by about 57% and 27% respectively. Moreover, *L. acidophilus* treated mice exhibited improved serum levels of IFN-γ, IL-10, CD4^+^ and CD8^+^ cells, manifesting a better protective effects (108).

As a growing number of therapeutic targets have been identified, the idea of genetically engineering bacteria to combat a specific pathogenic process is gaining much attention. Indoleamine 2,3-dioxygenase (IDO), which is an immune check point protein contributing to the immunosuppressive TME, is related to the poor prognosis of colon cancer patients. Melstrom *et al.* successfully reduced the IDO levels by employing *S. typhimurium* which delivers inhibitory small hairpin (sh)RNA targeting IDO. The treatment resulted in significant delayed tumor progression in CT26 and MC38 colon cancer models, where enhanced neutrophils infiltration was observed, indicating the innate immune response was efficiently elicited (109). The overwhelming activation of signal transduction and transcription activator 3 (Stat3) is reported to promote tumorigenesis *via* various mechanisms. By combining the inhibitor of Stat3 (nifuroxazide) with *S. typhimurium* carrying small interfering RNA against PD-1, Feng *et al.* discovered a synergistic antitumor effect on colon cancer, where potent anti-tumor immunity was strongly elicited (110).

### 5.2 Lung Cancer

It has been shown that a number of probiotics are blessed with anti-tumor efficacy through immunological regulation. To investigate the underlying mechanism, Ghaemi *et al.* demonstrated that intravenous injection of *B. bifidum* led to increased secretion of IFN-γ and IL-12, enhanced lymphocyte proliferation and CD8^+^ T cell responses as compared to oral administration in a HPV-induced TC-1 mouse lung cancer model (112). Similarly, by using the same cancer model, *L. casei* BL23 was discovered to exert anti-tumor effect by IL-2 signaling pathway, with the involvement of T cells and NK cells (113). Moreover, recombinant strain of *L. lactis* that secreted biologically active IL-17A cytokine was also established, which made 26% of treated mice tumor-free in the TC-1 tumor challenge (114).

To obtain vigorous anti-tumor immunity, simultaneously targeting both the costimulatory and inhibitory receptor-ligands of the immune system can be a promising strategy. In support of this idea, agonist antibody to glucocorticoid-induced tumor necrosis factor receptor-related protein (GITR) which acts as a costimulatory target that promotes effector function has been combined with a *L. monocytogenes*-based vaccine which significantly regulates the suppressive cells including Treg cells and MDSCs. In a mouse model bearing subcutaneous TC-1 lung tumor, this combined therapy resulted in tumor eradication in 60% of treated mice, which could be attributed to the enhanced function of CD8^+^ T cell, reduced ratio of Treg/CD4^+^ cell and the regulation to MDSCs (111).

### 5.3 Melanoma

Melanoma represents as the most aggressive type of skin cancer with a high tendency to progress into the metastatic stage, which may attribute to the notable competency to evade the immune recognition. Therefore, potentiating the immune attack against melanoma is a viable strategy. Poliseno *et al.* has reported that attenuated *L. monocytogenes* could kill various melanoma cells *in vitro*, regardless of their stage and genetic status, which may overcome therapeutic challenge caused by the high degree of heterogeneity. By establishing genetically engineered mice susceptible to primary and metastatic melanoma, the team further assessed the anti-tumor activity of this strain *in vivo*, which resulted in impaired growth of the primary tumor as well as reduction of the metastatic burden. Moreover, increased infiltration of CD4^+^ and CD8^+^ T cells was detected, suggesting that the immune responses were effectively augmented (115). To step further, *L. monocytogenes* expressing tumor-associated antigen was successfully constructed, which elicited profound CD8^+^ T cells responses and subsequently protected about 70% mice from B16F10 melanoma. When combined with immune checkpoint blockade therapy (anti-PD-1, anti-PD-L1, and anti-CTLA-4), significant tumor remission was observed, indicating that anti-tumor immunity induced by *L. monocytogenes* vaccination could be further enhanced with immune checkpoint blockade therapy (116). To better safeguard the application of live strains used in immunocompromised cancer patients, listeriolysin O, which is responsible for the biological activities of *L. monocytogenes* related to the anti-tumor effect, was encapsulated into gold nanoparticles to generate a safer preparation. Similarly, the ability of inducing CD8^+^ T cells responses was successfully maintained, and a synergism coupled with anti-PD-1 or anti-CTLA-4 was also detected (117).

### 5.4 Breast cancer

Only a few studies have reported the bacteria-based immunotherapeutic platforms targeting the unique characteristics in breast cancer. Min *et al.* has shown that *S. typhimurium* displaying the RGD peptide could specifically bind to cancer cells overexpressing αvβ3 integrin, including breast cancer cells. In a mouse model of human breast cancer (MDA-MB-231 cell line), significant tumor regression and prolonged survival of mice receiving intravenous injection of the modified strain were detected, in which the therapeutic effect relied largely on the tumor-specific accumulation following administration (61). Similarly, aptamers which promoted the colonization of bacteria inside tumor areas were also conjugated to *S. typhimurium* VNP20009 by Tan et al, which manifested excellent anti-tumor efficacy in 4T1 tumor-bearing mouse models. Moreover, the percentage of CD3^+^CD4^+^ T cells and the production of IFN- γ and TNF-α ware significantly elevated, suggesting strong immune response triggered by this bacterial agent (63). In another mouse model of triple negative breast cancer, impaired tumor growth and notable reduction in lung metastasis were reported in mice treated with *E. coli* encoding nanobody antagonist of CD47 (69).

### 5.5 Other Types of Cancers

Recently, immunotherapy, especially the immune checkpoint blockade therapy, has gained encouraging achievements in the treatment of lymphoma (123), and utilizing bacteria to delivery therapeutic antibody also attracts research attention. Danino *et al.* has investigated the anti-tumor activity of *E. coli* expressing both anti-PD-L1 and anti-CTLA-4 antibodies against advanced lymphoma in a mouse model with a larger initial volume (about 200 to 700 mm^3^), in which impaired growth or complete clearance was observed (67). Similarly, *E. coli* expressing CD47 antagonist was reported to exert durable anti-tumor efficacy of the established A20 tumors. Moreover, the treated mice obtained resistance when tumors cells were reinjected subcutaneously (69).

Inducing systemic immune responses by certain antigens overexpressed by cancer cells is a potent therapeutic method for metastatic cancer treatment. For instance, *S. typhimurium* carrying a plasmid encoding prostate stem cell antigen was successfully established, which induced Th1 immune responses and resulted in 50% of treated mice tumor-free over the challenge of TRAMPC1 mouse prostate cancer cells (118). Recombinant *L. monocytogenes* expressing HPV16-E7 was also demonstrated to generate protective effect in immunized mice against cervical cancer, where induction of Th1 immunity, enhanced lymphocyte proliferation and specific CTL activity were observed as compared to control group (119). Therefore, more therapeutic modalities targeting certain antigens displayed by specific tumors are yet to be further developed. For certain cancer type with low immunogenicity and low expression of neoantigen such as pancreatic ductal adenocarcinoma, Gravekamp *et al.* has designed a platform to deliver the immunogenic tetanus toxoid protein by *L. monocytogenes*. In this method, the tetanus toxoid acted as an alternative neoantigen to awaken the preexisting memory T cells generated in childhood vaccination, thus turning the cold TME into a highly immunological environment, which eventually resulted in over 80% reduction of tumor growth and metastasis when coupled with the treatment of gemcitabine (95).

## 6 Perspectives and Prospects

Different human body niches reside distinct microbiota communities and bacteria often perform different roles in different ecological sites. It is aware that bacteria being in the wrong place within the body can be quite hazardous. For example, *E. coli*, as a typical resident in the intestine, can cause infection once entering the urethra (124), abdominal cavity (125), and other regions of the body (126). Due to the in-depth understanding of the interaction between microorganisms and tumors, utilizing specialized bacteria for distinct tumor types can not only avoid infection, but also exert a regulatory influence on the local microecology. Shisssss *et al.* found that oral administration of *Akkermansia marcescens* can produce a synergistic effect with IL-2 therapy, and a good therapeutic effect was observed in a mouse model of colorectal cancer (127). Similarly, Zheng *et al.* reported that oral squamous cell carcinoma patients with higher levels of bacteria of the genus *Peptostreptococcus* presented higher probability of long-term survival. To upregulate the levels of *Peptostreptococcus*, subcutaneous injection of an adhesive hydrogel incorporating silver nanoparticles alongside the intratumoral delivery of the bacterium *P. anaerobius* was adopted, which manifested enhanced anti-tumor responses and synergized with the anti-PD-1 therapy. Therefore, In the future, commensal bacteria at different body sites can be specifically developed to exert therapeutic effects for the tumors at their according locus.

Currently, immune therapy exhibited immune-related adverse events (irAEs) such as colitis, fatigue, rash, endocrine disturbance, and hepatotoxicity (128, 129), which can be attributed to off-target effects of therapeutic drugs as well as dose-dependent toxicity. Relying on the precise regulation of the targeting properties of engineered bacteria, specific release of drugs at the tumor site can be achieved, which is beneficial to reduce the occurrence of adverse reactions related to immunotherapy. Besides, plenty of commensal probiotics such as Lactic acid bacteria, has exhibited benefits for the prevention of colitis and moderation of diarrhea, indicating that it is a promising choice to employ engineered probiotics to alleviate some adverse reactions (130).

**Figure 2:**
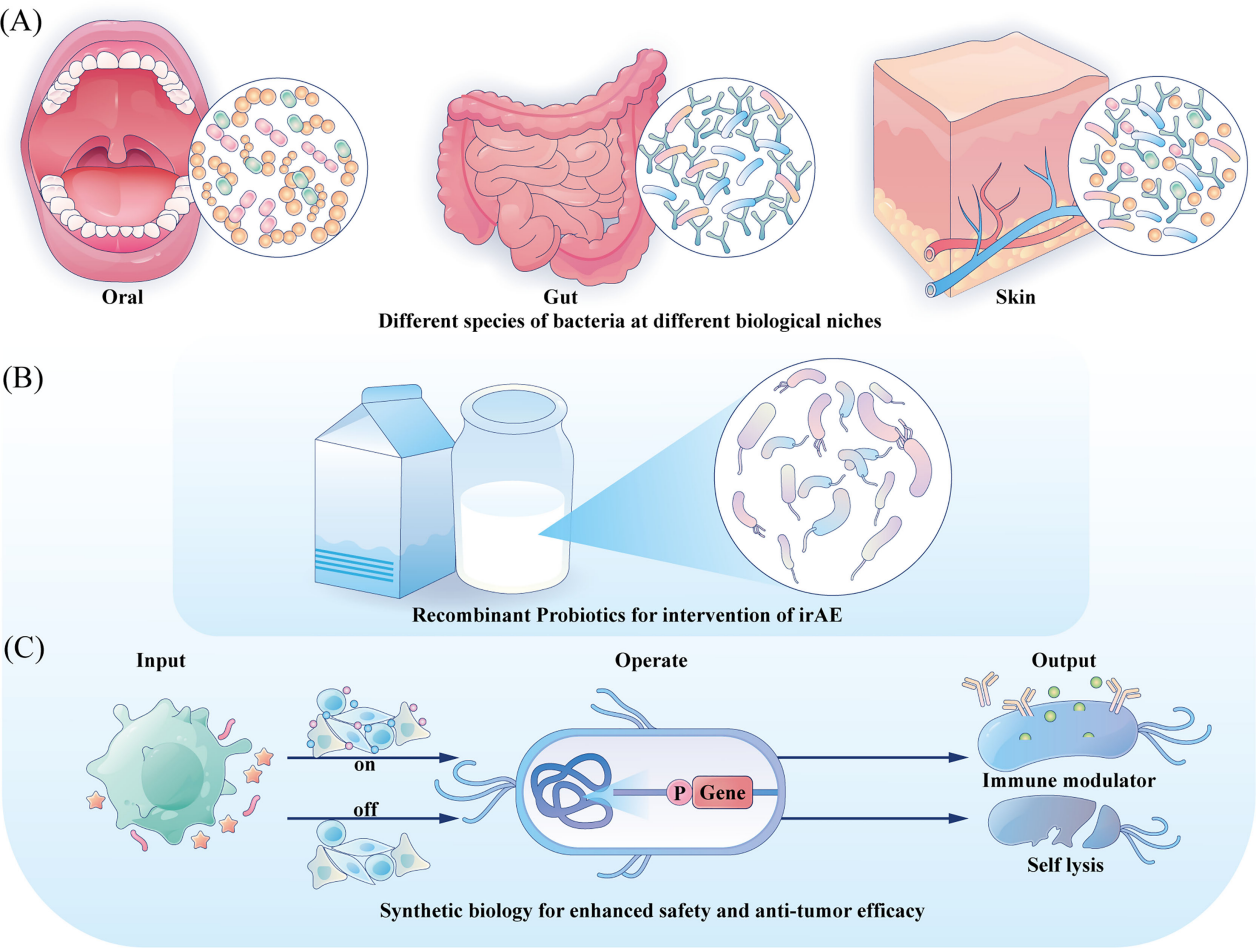
Directions for future engineering. **(A)** Engineering the commensal bacteria at their original ecology with enhanced anti-tumor prospects to provide precise treatment strategy. **(B)** For irAE, probiotics could be developed with anti-inflammation characteristic and serve as local mediators. **(C)** Integration gene circuits could manipulate the bacteria to sense different input information and provide different outputs, tuning the treatment intensity and controlling the bacteria fate.

Decades have passed since the first trial of utilizing BCG as medication for bladder cancer, and relentless practices have also been undergoing to investigate its involvement in cancer therapy beyond bladder cancer (131). Besides BCG, other bacterial preparations such as modifed *S. typhimurium* stains are also in the preclinical or clinical trial stage to better verify their safety and therapeutic effects (132). As bacteria are complex and viable therapeutic agents, some uncontrollable mutations during their proliferation may bring potential toxicity. And their inherent virulence can also lead to complex infections in immunocompromised cancer patients. However, the rapid advances in synthetic biology are making it possible to program a desired bacterial behavior through the introduction of synthetic gene circuits, which are composed of an input module detecting biotic signals, an operation module computing transmitted signal and an output module generating the desired cellular response, resulting in a safer application profile and enhanced anti-tumor efficacy (27). Directions for future engineering are illustrated in **Figure 2**.

## Author Contributions

QT and JC constructed figures and wrote the manuscript. LC revised the paper. All authors contributed to the article and approved the submitted version.

## Funding

This work was supported by the National Natural Science Foundation of China 81870759 (LC), 82071106 (LC), the Research Funding from West China School/Hospital of Stomatology Sichuan University, No. RCDWJS2022-5 (to JC), and the Research Funding from West China School/Hospital of Stomatology Sichuan University, No. RCDWJS2021-19 (to LC).

## Conflict of Interest

The authors declare that the research was conducted in the absence of any commercial or financial relationships that could be construed as a potential conflict of interest.

## Publisher’s Note

All claims expressed in this article are solely those of the authors and do not necessarily represent those of their affiliated organizations, or those of the publisher, the editors and the reviewers. Any product that may be evaluated in this article, or claim that may be made by its manufacturer, is not guaranteed or endorsed by the publisher.
